# Data of piezoelectric vibration energy harvesting of a bridge undergoing vibration testing and train passage

**DOI:** 10.1016/j.dib.2018.01.009

**Published:** 2018-01-28

**Authors:** Paul Cahill, Budhaditya Hazra, Raid Karoumi, Alan Mathewson, Vikram Pakrashi

**Affiliations:** a Centre for Marine and Renewable Energy Ireland (MaREI), Environmental Research Institute, University College Cork, Beaufort Building, Ringaskiddy, Co. Cork, Ireland; b Department of Civil Engineering, Indian Institute of Technology Guwahati, Guwahati, Assam 781039, India; c Department of Civil & Architectural Engineering, Royal Institute of Technology (KTH), Brinellvãgen 23, 10044 Stockholm, Sweden; d Heterogeneous Systems Integration, Micro & Nano Systems, Tyndall National Institute, Dyke Parade, Cork, Ireland; e Dynamical Systems and Risk Laboratory, School of Mechanical and Materials Engineering and Centre for Marine and Renewable Energy Ireland (MaREI), University College Dublin, Belfield, Dublin 4, Ireland; f Marine and Renewable Energy Ireland (MaREI) Centre, University College Dublin, Ireland

## Abstract

The data presented in this article is in relation to the research article “Vibration energy harvesting based monitoring of an operational bridge undergoing forced vibration and train passage” Cahill et al. (2018) [1]. The article provides data on the full-scale bridge testing using piezoelectric vibration energy harvesters on Pershagen Bridge, Sweden. The bridge is actively excited via a swept sinusoidal input. During the testing, the bridge remains operational and train passages continue. The test recordings include the voltage responses obtained from the vibration energy harvesters during these tests and train passages. The original dataset is made available to encourage the use of energy harvesting for Structural Health Monitoring.

**Specifications Table** *[please fill in right-hand column of the table below]*

**Table:** 

Subject area	*Structural Dynamics*
More specific subject area	*Energy Harvesting, Structural Health Monitoring, Bridge Engineering*
Type of data	*Figures, Excel Datasheet*
How data was acquired	*By deploying piezoelectric energy harvesters to a rail-bridge, while exciting the bridge on site using a shaker while allowing train passages.*
Data format	*Raw*
Experimental factors	*The swept sinusoidal excitation was from 3 to 50 Hz with 0.05 Hz/s rate with one exception of 5–10 Hz with 0.01 Hz/s rate with load amplitudes 5* *kN and 10* *kN respectively. The applied preloads were 15* *kN and 10* *kN. The natural frequencies of the cantilever piezoelectric energy harvesters using polyvinylidene fluoride (PVDF) material were 6.09 Hz, 7.11 Hz, 8.37 Hz, 15.75 Hz, 17.95 Hz and 20.45 Hz respectively.*
Experimental features	*Energy harvesting signatures recorded for different harvesters due to the response of the bridge related to the swept sinusoidal excitation and train passages.*
Data source location	*Södertälje*, *Sweden*
Data accessibility	*With this article*

**Value of the data**

• We expect this to be the first public domain dataset around energy harvesting based monitoring for bridges.
• The data will provide a benchmark for structural health monitoring researchers to overcome real challenges in site conditions, when coming up with methods for analysis or markers for monitoring.
• The data is expected to be an important resource for assessing and developing output-only system identification and monitoring algorithms.
• The data will serve as a key reference for future research in energy harvesting based structural health monitoring.

## 1 Data

The data provided here is related to deployment and monitoring of Pershagen Bridge, Sweden using piezoelectric energy harvesters [1] and is related to earlier studies on the concept of vibration energy harvesting based monitoring of built infrastructure [2], [3], [4]. A total of six energy harvesting devices with different natural frequencies designed around the natural frequency of the bridge are deployed and the bridge is tested using a shaker with swept sinusoidal loading with different frequency ranges and for different magnitudes of loads.

## 2 Experimental design, materials and methods

### 2.1 Cantilever piezoelectric energy harvester design

**Fig. 1:**
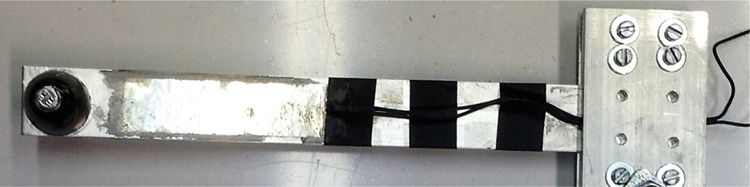
An example of a piezoelectric cantilever type energy harvester deployed in the bridge.

As the natural frequency of the bridge structure onto which the piezoelectric device is to be deployed is not precisely known, it is important to maximise the effective operational bandwidth of the energy harvester. As a result, six different cantilever type piezoelectric energy harvesters were chosen. The energy harvesting material was polyvinylidene fluoride (PVDF) sheets, which were bonded to the cantilever. The cantilever was attached to a rigid base. Fig. 1 presents an example.

The cantilevers could be tested in the laboratory full-scale using a permanent magnet shaker before being deployed on the bridge. The design allows for harvesters to be tuned to different natural frequencies. For the current test, these were 6.09 Hz, 7.11 Hz, 8.37 Hz, 15.75 Hz, 17.95 Hz and 20.45 Hz respectively. The key geometric properties of the cantilevers, along with their masses are presented in Table 1.

**Table 1:** Key geometric properties of the cantilever type piezoelectric energy harvesters and their masses.

**Parameter**	**Cant 1**	**Cant 2**	**Cant 3**	**Cant 4**	**Cant 5**	**Cant 6**
Length (m)	0.2195	0.2125	0.2545	0.1645	0.1775	0.151
Width (m)	0.0265	0.0265	00.031	0.0257	0.0256	0.0258
Thickness (m)	0.0015	0.0015	0.0015	0.0012	0.0012	0.0012
Mass (kg)	0.0692	0.0683	0.0663	0.0185	0.0191	0.0189

### 2.2 Device fabrication and assembly

The PVDF material was bonded to the aluminium cantilever. A 52-micron PVDF with silver electrodes was used. The material was cut to a size of 40 mm in length and 20 mm in width. Using copper conductive adhesive tape, two output solid core wires were attached to upper and lower electrodes to remove the output voltage and connected to a variable resistor to complete the circuit. The resistance was set to a constant value of 1 MΩ. A hole was created at one end of the cantilever through which a tip mass was placed and the PVDF harvester was bonded to the upper surface of the aluminium beam.

### 2.3 Details of host bridge structure

**Fig. 2:**
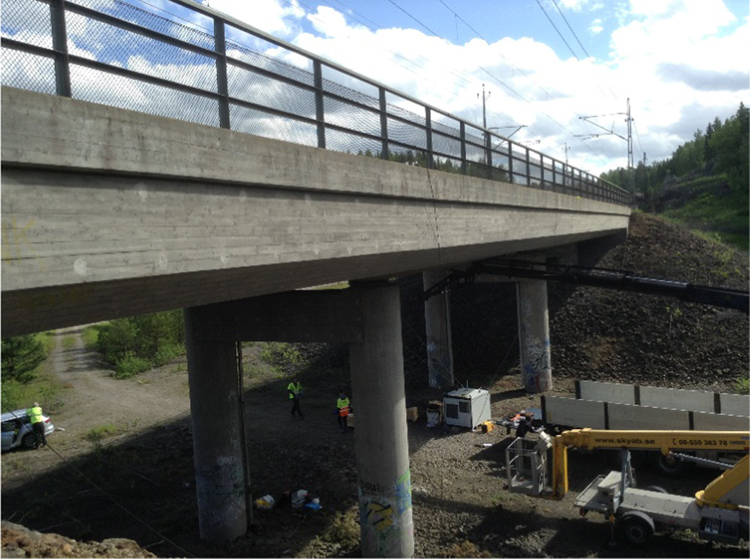
Photograph of Pershagen Bridge, Sweden used for testing a deployment of energy harvesters.

The host bridge was the Pershagen Bridge, Sweden (Fig. 2). The bridge is a 46.6 m long slab double track rail bridge, consisting of three spans and four supports. The central span is 18.8 m in length and the two side spans have a length of 11.1 m. An overhang exists between the side-spans and the abutments, which

rest on backfill embankments. The bridge is 11.9 m in width out to out and carries ballast of depth 0.6 m atop of the reinforced concrete slab deck, above which rests the train tracks.

### 2.4 Description of shaker unit

**Fig. 3:**
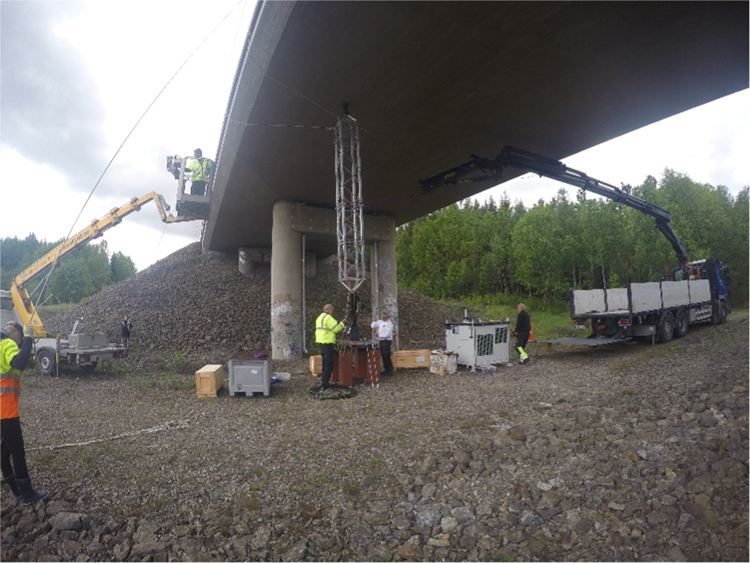
Photograph of shaker for providing excitation to Pershagen Bridge.

The hydraulic shaker unit (Fig. 3) used to excite the Pershagen Bridge is designed by the Division of Structural Engineering and Bridges at KTH Royal Institute of Technology, Sweden [5]. It consists of a hydraulic cylinder with an attached strut, atop of which is a load cell providing a feedback loop. This allows for the force, frequency and displacement of the load being applied to the bridge structure to be constantly maintained. The shaker has the ability to apply a swept sinusoidal loading of varying magnitudes to the connected bridge. As the shaker unit is designed to be positioned, and subsequently apply loadings from, below the structure, traffic over the bridge is unaffected for the duration of the dynamic testing. Issues related to disruption to services is thus not a factor for consideration.

### 2.5 Outline of test plan

The shaker unit was placed 2.4 m from the longitudinal midspan of the main central span and 3.45 m from the edge of the bridge. A preload was applied by the shaker between the ground and the bridge, to ensure constant contact between the two during dynamic testing. To determine the response of the bridge to such tests, an array of nine uni-axial accelerometers were mounted along its top edge beams, with data collected using a HBM MGCPlus data acquisition system at a sampling frequency of 600 Hz. A total of four sets of dynamical tests were completed on the bridge. The first of these tests was carried out as an initial assessment to ensure all systems were operating correctly and that appropriate loadings were being applied. The second and third set of tests were carried out at two different loading magnitudes with a similar frequency range and rate of loading applied for both. The fourth set of tests was conducted at a reduced loading rate over a narrow frequency range, centered about the estimated natural frequency. The loading details of the testing conditions are provided in Table 2.

**Table 2:** Details of applied loadings during dynamical testing of host bridge.

**Test**	**Applied pre-load**	**Load amplitude**	**Frequency range**	**Loading rate**
Test 1	15 kN	5 kN	3–50 Hz	0.05 Hz/s
Test 2	15 kN	10 kN	3–50 Hz	0.05 Hz/s
Test 3	15 kN	10 kN	5–10 Hz	0.01 Hz/s

### 2.6 Deployment of harvesters

The energy harvesting devices were installed close to the shaker unit and to an accelerometer. Proximity to the shaker unit increased the responses of harvesters due to higher dynamic responses of the bridge. The accelerometer provided a reference for base excitation inputs to the harvesters. These harvesters were affixed to the top edge beam of the bridge, with the accelerometer at the center. The devices were placed so that the cantilevers were overhanging the bridge, to prevent them meeting the deck or other items. The response of the devices was monitored and recorded typically at a sampling rate of 100 Hz.

## 3 Measurements of response and monitoring

**Fig. 4:**
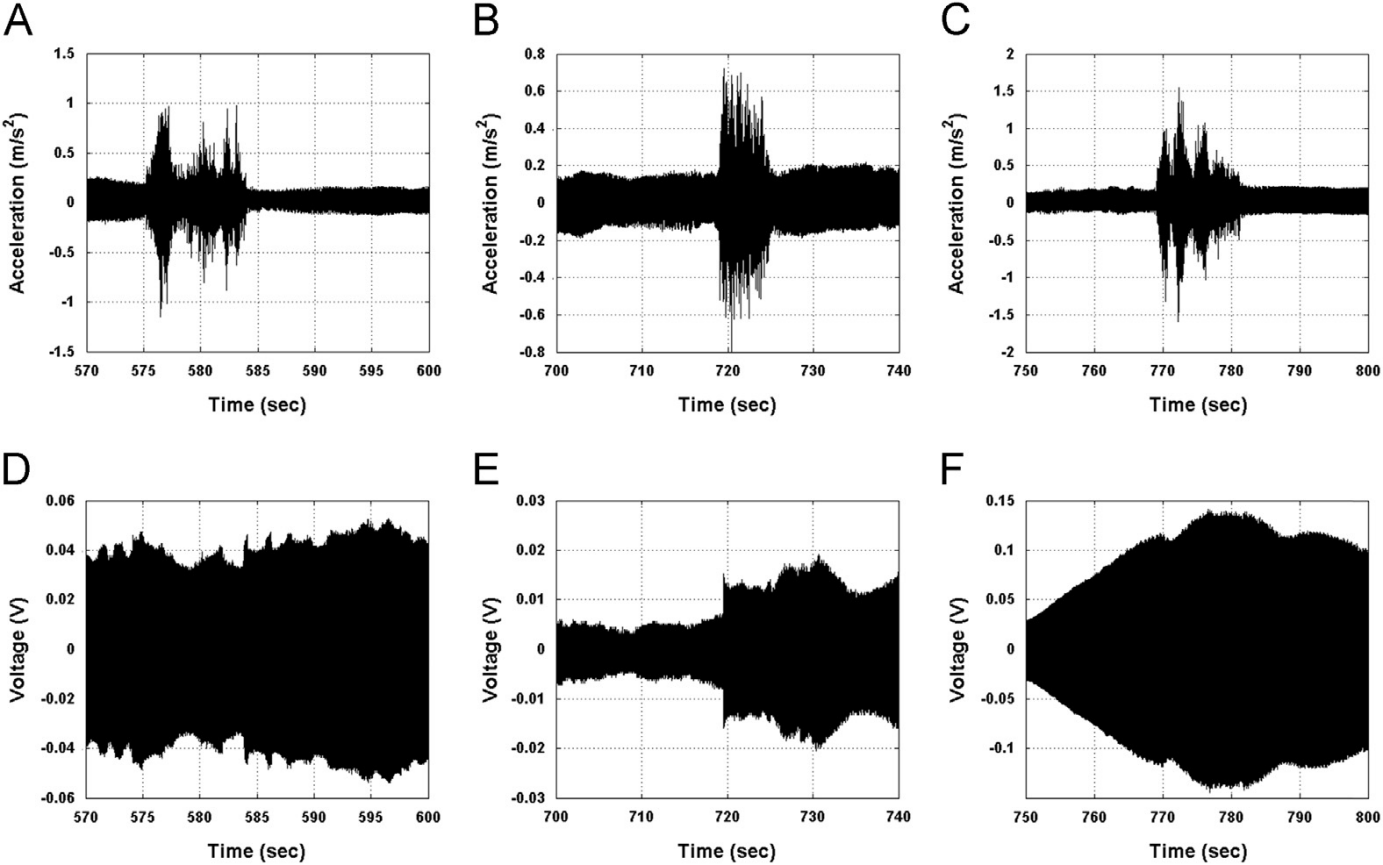
Acceleration and voltage responses from harvesters from Pershagen Bridge during swept sine testing and passage of trains.

**Fig. 5:**
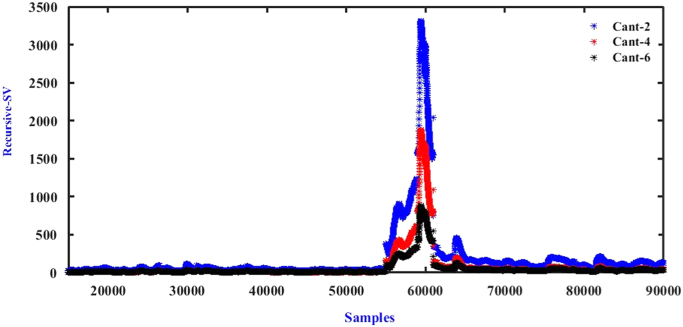
Recursive singular values for train passage detection.

Harvesting responses were recorded as voltages and compared against accelerometer responses. An example is presented in Fig. 4. Statistical indicators and algorithms [6], [7], [8] can subsequently be used to detect features of interest (Fig. 5).
